# Disability inclusion and community engagement: lessons from trachoma

**Published:** 2022-09-20

**Authors:** Adugna Amin, Juliana Amanyi-Enegela, Faizah Tosin Okunade, Tim Jesudason

**Affiliations:** Neglected Tropical Diseases Programme Manager: Light for the World, Addis Ababa, Ethiopia.; Neglected Tropical Diseases Programme Manager: CBM International, Cambridge, UK.; Neglected Tropical Diseases Programme Officer: CBM, Abuja, Nigeria.; Special Projects and Campaign Partnerships: International Coalition for Trachoma Control, London, UK.

**Globally, around one billion people live with disabilities, accounting for 15% of the global population.**

Understanding the needs of people with disabilities, and the barriers they face, is an essential first step towards ensuring they are included in eye health services, including trachoma elimination programmes.

In general, these barriers include a lack of access to information, the attitudes of health care workers to people with disabilities, the physically accessibility of health care facilities, and the affordability of services. Barriers can vary significantly according to the type of disability experienced, as well as the cultural and socioeconomic context, which is why it is critical that programmes collect disability disaggregated data, conduct baseline disability assessments, and systematically include people with disabilities throughout programme planning in order to tailor approaches.

In the Tigray region, Ethiopia, a baseline audit of the trachoma programme was conducted from 2018-2020 as part of an inclusive trachoma programme intervention initiative. The audit was carried out by Light for the World in partnership with the Ethiopian Centre for Disability and Development. It included a series of focus group discussions with communities, local officials, and eye care unit personnel to identify their knowledge, attitudes, and practices around implementing programmes that serve people with disabilities. It also included site visits to surgery outreach sites, mass drug administration (MDA) distribution points, and water, sanitation, and hygiene (WASH) facilities constructed by the trachoma programme. The audit documented a lack of knowledge among health workers, including trachomatous trichiasis (TT) surgeons, about how to implement programmes that are inclusive for people with disabilities.

**Figure:**
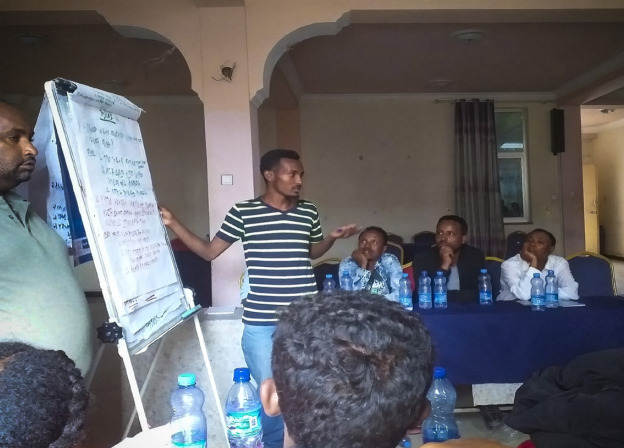
Disability awareness training in Wukro Town. ethiopia

Recognising that increased efforts were needed, implementing partners worked with organisations of people with disabilities to develop sign language manuals and an image toolbox. The sign language manuals help service providers to communicate with deaf persons, while the image toolbox provides materials to educate persons with intellectual disabilities about the disease.

Representatives from organisations of people with disabilities were involved in the planning of MDA activities. This resulted in several changes: a two-hour training session on disability inclusion for the MDA team, collecting disability disaggregated data, identifying distribution points that are accessible to people with disabilities, and proactive efforts to identify people with disabilities who require home care to access interventions. These changes are now being included in all programmes for trachoma across Tigray.

Improving access for people with disabilities is particularly important in settings affected by conflict and humanitarian emergencies. In Jigawa State, North West Nigeria, surgical outreach campaigns have been adapted to take interventions closer to people in need, rather than hosting them at a central location. This has made the sites more accessible to people with disabilities, while also reducing the cost of attending outreach campaigns for those living in remote areas.

Another development is the recruitment of TT case finders and TT surgeon assistants to ensure that patients are being found, followed up, and appropriately managed. This is particularly important for people with disabilities who face cost and mobility access barriers when having to travel. Case finders are employed to identify people who need TT surgery, and TT surgeon assistants then provide surgical management services in the closest facilities to the individual's home.

Experiences from the global trachoma programme demonstrate how tailored programming delivers integrated people-centred eye care and ensures accessibility of services, as recommended by the World Health Organization's World Report on Vision.^1^ To achieve global elimination of trachoma as a public health problem, as targeted by the global NTD road map, integration of case finding and TT surgery into routine eye health systems will be key. Trachoma programmes offer relevant lessons on ensuring programmes are inclusive, by training health workers about disability inclusion, and ensuring interventions (including rehabilitation), are accessible and affordable, so that no one is left behind across trachoma and wider eye health services.
